# The Effect of Estrogen Replacement Therapy on Alzheimer's Disease and Parkinson's Disease in Postmenopausal Women: A Meta-Analysis

**DOI:** 10.3389/fnins.2020.00157

**Published:** 2020-03-10

**Authors:** Yu-jia Song, Shu-ran Li, Xiao-wan Li, Xi Chen, Ze-xu Wei, Qing-shan Liu, Yong Cheng

**Affiliations:** Key Laboratory of Ethnomedicine of Ministry of Education, Center on Translational Neuroscience, School of Pharmacy, Minzu University of China, Beijing, China

**Keywords:** estrogen replacement therapy, Alzheimer's disease, Parkinson's disease, meta-analysis, systematic review

## Abstract

**Background:** Estrogen replacement therapy (ERT) is a common treatment method for menopausal syndrome; however, its therapeutic value for the treatment of neurological diseases is still unclear. Epidemiological studies were performed, and the effect of postmenopausal ERT on treating neurodegenerative diseases, including Alzheimer's disease (AD) and Parkinson's disease (PD), was summarized through a meta-analysis.

**Methods:** Twenty-one articles were selected using a systematic searching of the contents listed on PubMed and Web of Science before June 1, 2019. Epidemiological studies were extracted, and relevant research data were obtained from the original articles based on the predefined inclusion criteria and data screening principles. The Comprehensive Meta-Analysis Version 2 software was used to pool effective size, test heterogeneity, conduct meta-regression and subgroup analysis, and to calculate publication bias.

**Results:** Our results showed that ERT significantly decreased the risk of onset and/or development of AD [odds ratio (OR): 0.672; 95% CI: 0.581–0.779; *P* < 0.001] and PD (OR: 0.470; 95% CI: 0.368–0.600; *P* < 0.001) compared with the control group. A subgroup and meta-regression analysis showed that study design and measure of effect were the source of heterogeneity. Age, sample size, hormone therapy ascertainment, duration of the treatment, or route of administration did not play a significant role in affecting the outcome of the meta-analysis.

**Conclusion:** We presented evidence here to support the use of estrogen therapy for the treatment of AD and PD.

## Introduction

Neurodegenerative diseases, such as Alzheimer's disease (AD) and Parkinson's disease (PD), are characterized by the sustained cell cycle arrest and production of a continuous senescence-associated secretory phenotype due to structural and functional changes in neurons (Kritsilis et al., 2018). According to global epidemiological data, between 2000 and 2013, death from AD increased by 71% (Prince et al., 2013). Next to AD in terms of incidence, PD is the second most common neurodegenerative disease and is characterized by the progressive damage of mesencephalic dopaminergic (DA) neurons of the substantia nigra (SN) and the striatal projections. The prevalence rate of PD was 100–200 per 100,000 people, and the annual incidence was 15 per 100,000 people in the United States (Ascherio and Schwarzschild, 2016). Neurodegenerative diseases often persist in the brain, making their pathogenesis difficult to study. Thus, it is urgent to develop effective prevention and treatment methods for the disease.

Some researches indicated that the risk of AD development and the severity differed significantly between men and women. The incidence of AD was two to three times higher among women than men, and premature menopause would increase the risk of onset and/or developing AD (Pike, 2017). PD was consistently observed to occur at a lesser frequency in women than in men at an approximate ratio of 1:1.5. During the progression of PD, female patients were usually associated with a more benign phenotype, suggesting the possible beneficial effect of estrogen (Picillo et al., 2017). The data suggest that perimenopause may increase the patient's vulnerability of developing neurological diseases, thus it may be a good window to perform menopausal hormone therapy for beneficial effects on patient's cognitive function.

A number of research reviews and *in vivo* and *in vitro* experiments with meta-analysis have been conducted to normalize clinical data due to individual differences in the link between estrogen replacement therapy (ERT) and its treatment effect on AD and PD. Several studies demonstrated that AD-related cognitive decline was improved and a lower risk of onset and/or developing AD was observed following the menopausal hormone therapy (Hogervorst et al., 2000; Bagger et al., 2005; Yesufu et al., 2007). However, controversial results have been reported. Other studies did not show significant differences between ERT and AD (Yaffe et al., 1998; Mulnard et al., 2000). Moreover, two studies advised that ERT should not be used for AD prevention (Shumaker et al., 2003; O'Brien et al., 2014). For PD, some investigations showed that there was an association between postmenopausal ERT and a lower risk of PD (Ragonese et al., 2004), while others did not observe such association (Rugbjerg et al., 2013). There has been no systematic meta-analysis for the connection between ERT and the risk of onset and/or developing PD.

In general, previous reviews relied mainly on qualitative analyses. The existing meta-analysis cannot reach a unified conclusion on whether there is a correlation between ERT, AD, and PD. Therefore, to address the inconsistent data, we included relevant scientific data prior to June 2019 and conducted a systematic and comprehensive analysis of the relationship between ERT and the risk of onset and/or developing AD and PD.

## Methods

### Study Selection and Data Collection

Relevant foreign literature was searched by two independent researchers from databases including PubMed and Web of Science. The following keywords were used as search input: estrogen therapy, ERT, hormone therapy, hormone replacement therapy, Alzheimer's disease, AD, Parkinson's disease, and PD. There was no year restriction applied. Additional articles were selected from the reference section of certain publications. Only full-text journal articles with accessible data for analysis were included.

**Figure 1:**
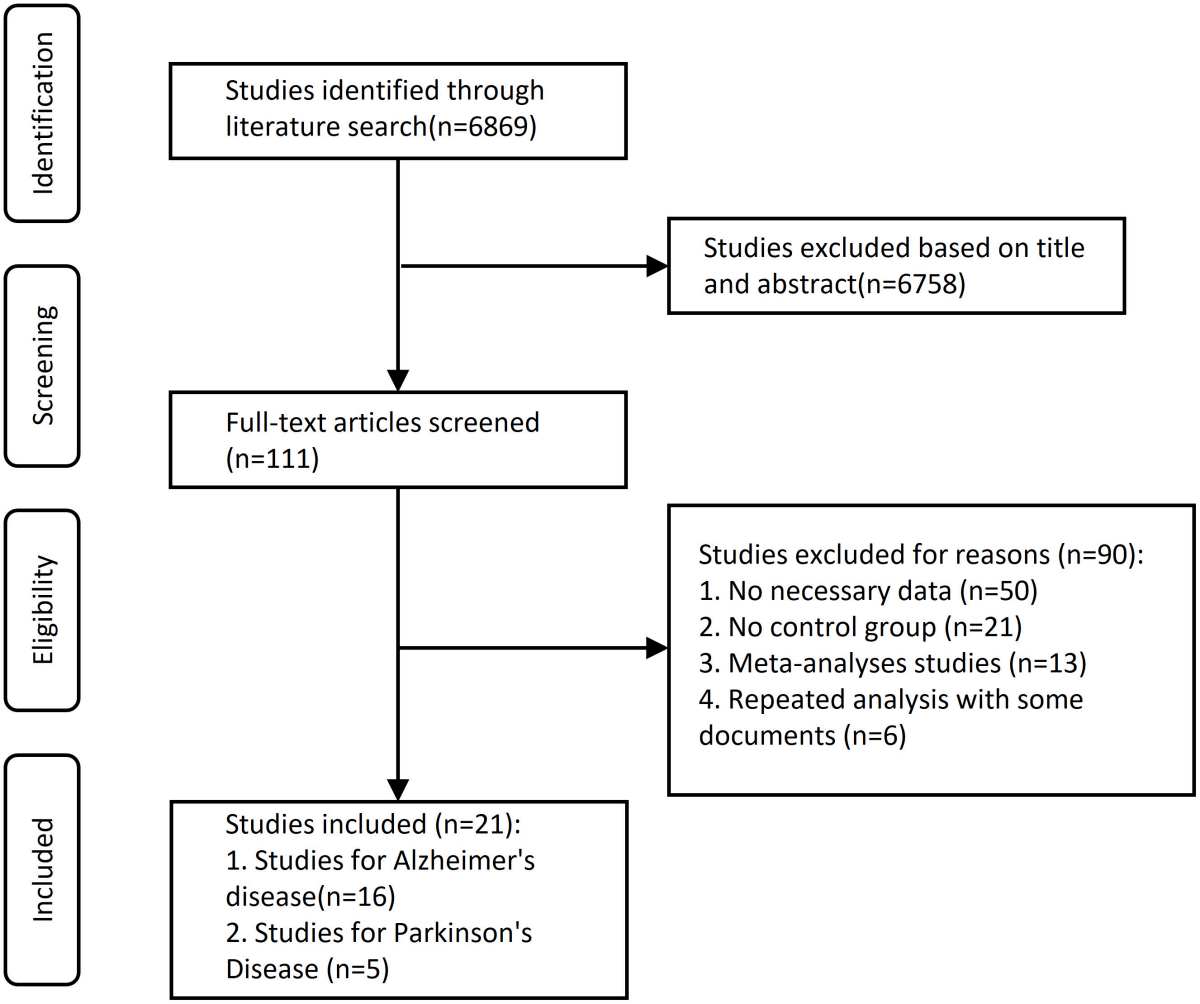
Flowchart describing the approach used to identify eligible studies. We conducted a systematic search on Medline (via PubMed and Web of Science) and covering all articles up until June 1, 2019.

The initial search yielded 3,668 records from PubMed and 3,201 records from Web of Science. After the screening of titles and abstracts, 6,758 records were excluded because they were not related to our present subject. The remaining 111 articles were selected for full-text scrutiny. Ninety studies were excluded due to no usable data (*n* = 50), no control group (*n* = 21), meta-analyses studies (*n* = 13), or repeated analysis with some documents (*n* = 6). Therefore, a total of 21 studies with 1,266 patient cases and 3,845 control cases were included in this meta-analysis. A flowchart of the selection process was presented in Figure 1.

### Data Extraction

For each selected study, the following data were extracted: study design, number of participants, number of AD case, number of control case, participants' ages, method for collecting data on hormone use, follow-up time, year of publication, measure of effect, diagnostic criteria, classifications and frequencies of hormone therapy application (e.g., timing of use, duration of use, route of administration, formulation, or any available information) and model, or other covariates.

Age was provided in the form of the mean value, unless otherwise stated. Nearly all the studies included adjusted odds ratio (OR)/relative risk (RR)/hazard risk (HR) values because there were some differences in covariates among the studies.

### Statistical Analysis

The Comprehensive Meta-Analysis Version 2 software (Biostat, Englewood, NJ, USA) was used for all the statistical analyses. We grouped study findings on the basis of how hormone therapy was categorized (e.g., any vs. never used) and included a summary for measure of effect and 95% CIs in the tables. These summaries were calculated based on random-effects models which involved a weighting scheme.

Cochran Q test was applied to evaluate the statistical difference of heterogeneity across different studies. It was considered statistically significant when *P* < 0.05. I^2^ index was used to determine the inconsistency across different studies to evaluate the impact of heterogeneity. We used 25, 50, and 75% of I^2^ to define low, medium, and high levels of heterogeneity. The Egger's test was used to determine the significance of a statistical test for publication bias to assess the degree of asymmetry in the funnel plot.

Meta regression was conducted among factors that might lead to heterogeneity in order to identify the main factors. A predefined subgroup analysis was used to assess the impact of various factors in the study. The following subgroups were defined in the AD group: case >500 vs. case ≤500, case-control study vs. prospective cohort, publishing year ≤1995 vs. 1996–2005 vs. 2006–2019, women age ≤70 vs. 71–79 vs. age ≥80, measure of effect: OR vs. HR vs. RR, hormone therapy ascertainment by interview vs. questionnaires vs. prescription database vs. medical records, duration of the treatment <5 years vs. 5–10 years vs. treatment >10 years. It was considered as statistically significant if *P* < 0.05. Meanwhile, the change in I^2^ was compared before and after the introduction of covariates into the regression model.

## Results

### Characteristics of Included Studies

We found 111 potentially relevant articles. Among these articles, a total of 21 eligible studies were pooled together for analyses (Figure 1). The Newcastle–Ottawa Scale (NOS) scores of eligible articles were between 7 and 8, with an average of 7.57. Baseline characteristics of included studies are shown in Tables 1–3.

**Table 1:** Characteristics of studies included.

**References**	**Country**	**Study design**	**Type of disease**	**No**.	**Start time**	**Interval over which disease was assessed**	Age^*^	**Hormone therapy ascertainment**	**Diagnostic criteria**	Effect measure^**^	**NOS score**
Broe et al., 1990	Australia	A case-control study	AD	170	1986	1986–1988	79	Interview	NINCDS-ADRDA	OR	8
Graves et al., 1990	America	A case-control study	AD	260	1980	1980–1985	64.9	Questionnaires	NINCDS-ADRDA	OR	8
Brenner et al., 1994	America	A case-control study	AD	227	1987	1987–1992	77.59	Prescription database	DSM-III-R, NINCDS-ADRDA	OR	8
Paganini-Hill and Henderson, 1994	America	A case-control study	AD	355	1981	1981–1992	86.74	Questionnaire	NINCDS-ADRDA	OR	8
Mortel and Meyer, 1995	America	A case-control study	AD	241	NR	NR	73.2	Medical records	DSM-III-R, NINCDS-ADRDA	OR	7
Tang et al., 1996	America	Prospective cohort	AD	1,124	NR	5 years	74.2	Prescription database	DSM-III-R, NINCDS-ADRDA	RR	7
Kawas et al., 1997	America	Prospective cohort	AD	472	1978	16 years	61.5	Multidisciplinary evaluations	DSM-III-R, NINCDS-ADRDA	RR	7
Slooter et al., 1999	Netherlands	A case-control study	AD	228	1980	1980–1987	58.06	Questionnaires	NINCDS-ADRDA	OR	8
Waring et al., 1999	America	A case-control study	AD	444	1980	1980–1984	82	Medical records	DSM-III-R, NINCDS-ADRDA	OR	7
Seshadri et al., 2001	United Kingdom	A case-control study	AD	280	1990	1990–1998	65.52	Prescription database	NINCDS-ADRDA	OR	8
Lindsay et al., 2002	America	Prospective cohort	AD	2,079	1991	1991–1996	73.3	Questionnaires	DSM-IV, NINCDS-ADRDA	OR	8
Zandi et al., 2002	America	Prospective cohort	AD	1,866	1998	1998–2000	74.4	Interview	NINCDS-ADRDA	HR	7
Henderson et al., 2005	America	A case-control study	AD	971	NR	6 months	50	Medical records	NR	OR	7
Roberts et al., 2006	America	A case-control study	AD	486	1985	1985–1989	84	Medical records	DSM-IV, NINCDS-ADRDA	OR	8
Lau et al., 2010	America	Cross-sectional study	AD	4,087	2005	2005–2007	77.1	Questionnaires	NPI-Q	OR	8
Shao et al., 2012	America	Prospective cohort	AD	1,768	1995	1995–2006	74.6	Questionnaires	NINCDS-ADRDA	HR	8
Imtiaz et al., 2017	Finland	A case-control study	AD	8,195	1999	1999–2009	72.3	Questionnaires	DSM-IV, NINCDS-ADRDA	HR	8
Fernandez and Lapane, 2000	America	Cross-sectional study	PD	10,145	1992	1992–2005	65	Medical records	MDS-UPDRS	OR	8
Martignoni et al., 2003	Italy	A case-control study	PD	442	NR	8.7 years	66.57	Questionnaires	MDS-UPDRS	Mean (SD)	7
Currie et al., 2004	America	A case-control study	PD	140	1999	NR	68.43	Interview	MDS-UPDRS	OR	7
Nicoletti et al., 2007	NR	Cross-sectional study	PD	11	NR	14 weeks	68.4	Clinical observation	MDS-UPDRS	Mean (SD)	7
Park et al., 2018	America	A case-control study	PD	300	2006	2006–2013	68.7	Questionnaires	MDS-UPDRS	OR	8

*NR, not reported; OR, odds ratio; RR, relative risk; HR, hazard ratio; AD, Alzheimer's disease; PD, Parkinson's disease; NINCDS-ADRDA, National Institute of Neurological and Communicative Disorders and Stroke-Alzheimer's Disease and Related Disorders Association; DSM-III-R, Diagnostic and Statistical Manual of Mental Disorders, Third Edition, Revised; DSM-IV, Diagnostic and Statistical Manual of Mental Disorders, Fourth Edition; MDS-UPDRS, Movement Disorder Society-Sponsored Revision Unified Parkinson's Disease Rating Scale; NOS, Newcastle–Ottawa Scale*.

* *The age provided is the value of the mean, unless otherwise stated*.

** *Nearly all studies included adjusted OR/RR values because there were some differences in covariates among the studies*.

**Table 2:** Summary of results–estrogen replacement therapy and Alzheimer's disease risk.

**References**	**Study design**	**No**.	**Covariates**	**Sample size (AD/Con)**	**OR/RR/HR**	**95% CI**	**Outcome**
Broe et al., 1990	A case-control study	170	Age, sex	11/24	0.34	0.12–0.94	Identified four risk factors for AD, there is no estrogen treatment.
Graves et al., 1990	A case-control study	260	NR	52/58	1.1	0.60–1.80	No statistically significant differences were observed between the two groups.
Brenner et al., 1994	A case-control study	227	Education, marital status, ethnicity, smoking or progestogen use	0/18	0.78	0.39–1.56	Provide no evidence that estrogen replacement therapy has an impact on the risk of Alzheimer's disease in women.
Paganini-Hill and Henderson, 1994	A case-control study	355	Age, weight, stroke, blood pressure, medication use	23/21	1.15	0.50–2.64	The increased incidence of Alzheimer's disease in older women may be due to estrogen deficiency and that it may be useful for preventing or delaying dementia.
Mortel and Meyer, 1995	A case-control study	241	Age	87/192	0.70	0.51–0.95	ERT may eventually prove to be a useful prophylactic agent for reducing risk of DAT and IVD among postmenopausal women.
Tang et al., 1996	Prospective cohort	1,124	Education, ethnicity, Apo E genotype	28/137	0.67	0.38–1.17	Estrogen use in postmenopausal women may delay the onset and decrease the risk of Alzheimer's disease.
Kawas et al., 1997	Prospective cohort	472	Age, education, age at menarche/menopause	9/221	0.46	0.21–0.99	Support for a protective influence of estrogen in AD.
Slooter et al., 1999	A case-control study	228	Age, education, Apo E genotype	372/324	0.53	0.39–0.73	Estrogen use is beneficial to Alzheimer's disease with early onset.
Waring et al., 1999	A case-control study	444	Age, education	4/121	1.37	0.48–3.95	Estrogen replacement therapy is associated with a reduced risk of AD in postmenopausal women.
Seshadri et al., 2001	A case-control study	280	Age, smoking, BMI, physician's practice	10/29	1.82	0.86–3.84	The use of HRT in women after the onset of menopause was not associated with a reduced risk of developing AD.
Lindsay et al., 2002	Prospective cohort	2,079	Age, education	28/25	1.10	0.63–1.93	No statistically significant association was found for estrogen replacement therapy can reduce risk of Alzheimer's disease.
Zandi et al., 2002	Prospective cohort	1,866	Age, education, Apo E genotypes	15/53	1.18	0.59–2.37	Prior HRT use is associated with reduced risk of AD, but there is no apparent benefit with current HRT use unless such use has exceeded 10 years.
Henderson et al., 2005	A case-control study	971	Age, education, race	87/1018	0.80	0.58–1.09	HT may protect younger women from AD or reduce the risk of early onset forms of AD, or that HT used during the early postmenopause may reduce AD risk.
Roberts et al., 2006	A case-control study	486	Age at menarche/ menopause, type of menopause, duration of fertile life, hypertension, diabetes, smoking, nonsteroidal anti-inflammatory drugs, education	9/148	0.50	0.25–0.90	Do not confirm a significant association between ET and AD.
Lau et al., 2010	Cross-sectional study	4,087	Age, sex, ethnicity, education, marital status and living arrangement	211/202	0.48	0.22–1.01	Number of medications used is associated with PIRx among ADC's community-dwelling elderly patients with and without dementia, polypharmacy increasing the risk of PIRx.
Shao et al., 2012	Prospective cohort	1,768	Education, alcohol or tobacco use, self-rated health status.	26/1038	0.59	0.36–0.96	Although possibly beneficial if taken during a critical window near menopause, HT initiated in later life may be associated with increased risk.

*NR, not reported; OR, odds ratio; RR, relative risk; HR, hazard ratio; AD, Alzheimer's disease; Con, control group; Apo E, apolipoprotein E; BMI, body mass index; DAT, dementia of the Alzheimer's type; IVD, ischemic vascular dementia; HRT, hormone replacement therapy; HT, hormone therapy; ET, estrogen therapy; PIRx, potentially inappropriate prescription medication; ADC, Alzheimer's disease center*.

**Table 3:** Summaryof results–estrogen replacement therapy and Parkinson's disease risk.

**References**	**Study design**	**No**.	**Covariates**	**Sample Size (PD/Con)**	**OR/RR/HR**	**95% CI**	**Outcome**
Fernandez and Lapane, 2000	Cross-sectional study	10,145	Age, race, and motor impairment	23/96	NR	NR	Shorter duration of estrogen use was associated with a modestly increased risk of Alzheimer's disease, and longer duration with a weakly decreased risk.
Martignoni et al., 2003	A case-control study	442	Age, mode, premenopausal menstrual irregularities, presence of climacteric symptoms	55/78	0.52	0.30–0.92	Estrogen's potential beneficial effects on PD motor and cognitive functions.
Currie et al., 2004	A case-control study	140	Age	4/6	0.99	0.27–3.57	The existence of a qualitative relationship between PD and reproductive events.
Nicoletti et al., 2007	Cross-sectional study	11	NR	17/36	0.33	0.16–0.68	Postmenopausal estrogen therapy may be associated with a reduced risk of PD in women.
Park et al., 2018	A case-control study	300	Ethnicity, education, smoking duration, disease	195/NR	0.475	0.31–0.72	Estrogen replacement therapy has a possible benefit on dyskinesias in postmenopausal women with PD.

*NR, not reported; OR, odds ratio; RR, relative risk; HR, hazard ratio; PD, Parkinson's disease; Con, control group*.

Stratification of the study: 13 were case-control studies, five were prospective cohort studies, and three were cross-sectional studies. Stratification of the location: 15 studies were conducted in America, three in Europe (one in the UK, one in Italy, and one in Netherlands), and two other countries (one in Canada, one in Australia). In the AD group, there were 13 studies in America, two in Europe (one in the UK and one in Netherlands), and one in other countries. Stratification of neurological disorders: five cases evaluated the impact of ERT on PD and 16 cases on AD. All studies were collected on the use of hormone therapy either by self-report (e.g., interview or questionnaire) at the start of the study, by electronic prescription database, or by medical records. Furthermore, all studies were included in this review except one reported using standard criteria to diagnose AD and dementia [e.g., National Institute of Neurological and Communicative Disorders and Stroke-Alzheimer's Disease and Related Disorders Association (NINCDS-ADRDA); Diagnostic and Statistical Manual of Mental Disorders, Third Edition, Revised (DSM-III-R); Diagnostic and Statistical Manual of Mental Disorders, Fourth Edition (DSM-IV); or Movement Disorder Society-Sponsored Revision Unified PD Rating Scale (MDS-UPDRS)].

### Association of AD and PD With ERT

**Figure 2:**
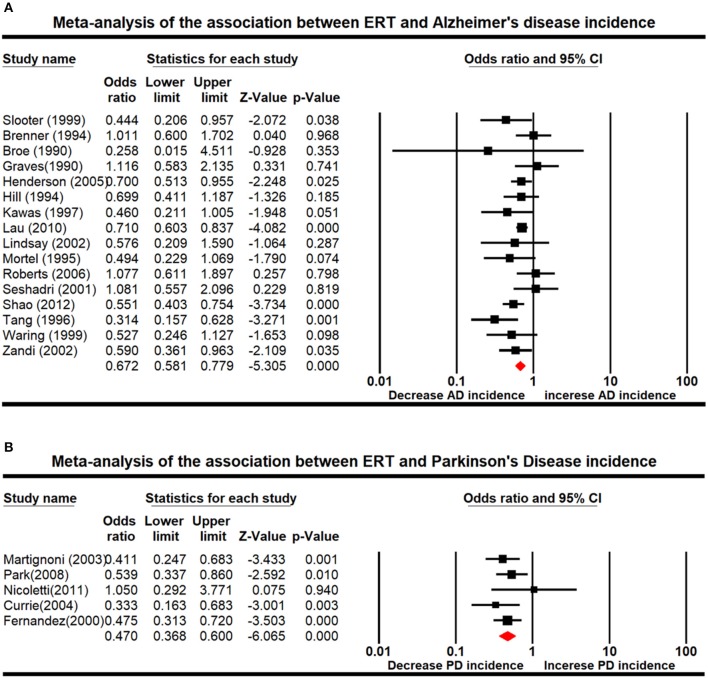
Forest plot displaying random-effects meta-analysis results for the association between Alzheimer's disease (AD) **(A)** and Parkinson's disease (PD) **(B)** and estrogen replacement therapy (ERT).

We used random-effects meta-analysis to assess the association between ERT and neurological diseases. Our results showed that ERT decreased risks of developing AD (OR: 0.672; 95% CI: 0.581–0.779; *P* < 0.001) and PD (OR: 0.470; 95% CI: 0.368–0.600; *P* < 0.001) in patients compared with the control (Figure 2), suggesting that estrogen therapy had a greater impact on PD.

### Investigation of Heterogeneity

Further subgroup analyses by disease outcome, 16 studies also had small heterogeneity in AD (*I*^2^ = 24.140; *P* = 0.181), but five studies of PD showed no heterogeneity (*I*^2^ = 0.000; *P* = 0.558). Since the data of PD were not enough, we only performed meta-regression analysis on the AD group. Study design (*P* = 0.01) and effect measure (*P* = 0.03) might be the sources of heterogeneity in the AD group, but number of cases (*P* = 0.172), age (*P* = 0.986), publication year (*P* = 0.712), hormone therapy ascertainment (*P* = 0.494), and duration of the treatment (*P* = 0.217) had no moderating effects on the significant association between hormone replacement therapy (HRT) and AD incidence (*P* > 0.05 in these studies) (Table 4).

**Table 4:** Summary of the subgroups analysis results.

**Analysis**	***N***	**Fix-effects model**		**Random-effects model**		**Heterogeneity**		**Meta regression**
		**OR (95% CI)**	***P***	**OR (95% CI)**	***P***	**I^**2**^ (%)**	***P***	***P***
All studies of AD	16	0.681 (0.612–0.759)	0.000	0.672 (0.581–0.779)	0.000	24.140	0.181	–
All studies of PD	5	0.470 (0.368–0.600)	0.000	0.470 (0.368–0.600)	0.000	0.000	0.558	–
**Subgroup 1 in AD group**								
Case > 500	6	0.653 (0.577–0.740)	0.000	0.627 (0.528–0.744)	0.000	26.755	0.234	0.17045
Case ≤ 500	10	0.771 (0.623–0.955)	0.017	0.758 (0.594–0.967)	0.026	19.734	0.261	
**Subgroup 2 in AD group**								
Case-control study	10	0.767 (0.641–0.920)	0.004	0.770 (0.633–0.936)	0.009	9.109	0.358	0.00867
Prospective cohort	5	0.519 (0.413–0.653)	0.000	0.519 (0.413–0.653)	0.000	0.000	0.635	
**Subgroup 3 in AD group**								
Year ≤ 1995	5	0.816 (0.607–1.096)	0.177	0.814 (0.602–1.101)	0.183	2.710	0.391	0.71219
1996–2005	8	0.608 (0.497–0.742)	0.000	0.592 (0.468–0.749)	0.000	17.281	0.294	
2006–2019	3	0.692 (0.601–0.797)	0.000	0.699 (0.534–0.915)	0.009	55.305	0.107	
**Subgroup 4 in AD group**								
Age ≤ 70	5	0.725 (0.574–0.915)	0.007	0.727 (0.527–1.003)	0.052	33.396	0.199	0.98581
Age 71–79	7	0.658 (0.578–0.749)	0.000	0.621 (0.500–0.770)	0.000	38.876	0.133	
Age ≥ 80	3	0.744 (0.548–1.093)	0.145	0.769 (0.524–1.12)	0.180	17.911	0.296	
**Subgroup 5 in AD group**								
Measure = OR	14	0.733 (0.650–0.827)	0.000	0.733 (0.650–0.827)	0.000	0.000	0.485	0.02941
Measure = HR	2	0.562 (0.432–0.732)	0.000	0.562 (0.432–0.732)	0.000	0.000	0.819	
Measure = RR	2	0.372 (0.221–0.624)	0.000	0.372 (0.221–0.624)	0.000	0.000	0.473	
**Subgroup 6 in AD group**								
Treatment < 5 Y	6	0.707 (0.619–0.808)	0.000	0.707 (0.619–0.808)	0.000	0.000	0.593	0.21689
Treatment 5–10 Y	6	0.745 (0.565–0.983)	0.037	0.705 (0.455–1.094)	0.119	58.180	0.035	
Treatment > 10 Y	3	0.571 (0.443–0.737)	0.000	0.571 (0.443–0.737)	0.000	0.000	0.637	
**Subgroup 7 in AD group**								
Interview	2	0.576 (0.355–0.934)	0.025	0.576 (0.355–0.934)	0.025	0.000	0.577	0.49442
Questionnaires	6	0.678 (0.593–0.775)	0.000	0.672 (0.572–0.788)	0.000	9.612	0.354	
Prescription database	3	0.762 (0.535–1.084)	0.130	0.714 (0.340–1.496)	0.372	76.373	0.015	
Medical records	4	0.711 (0.557–0.907)	0.006	0.709 (0.534–0.942)	0.018	14.937	0.317	

**Figure 3:**
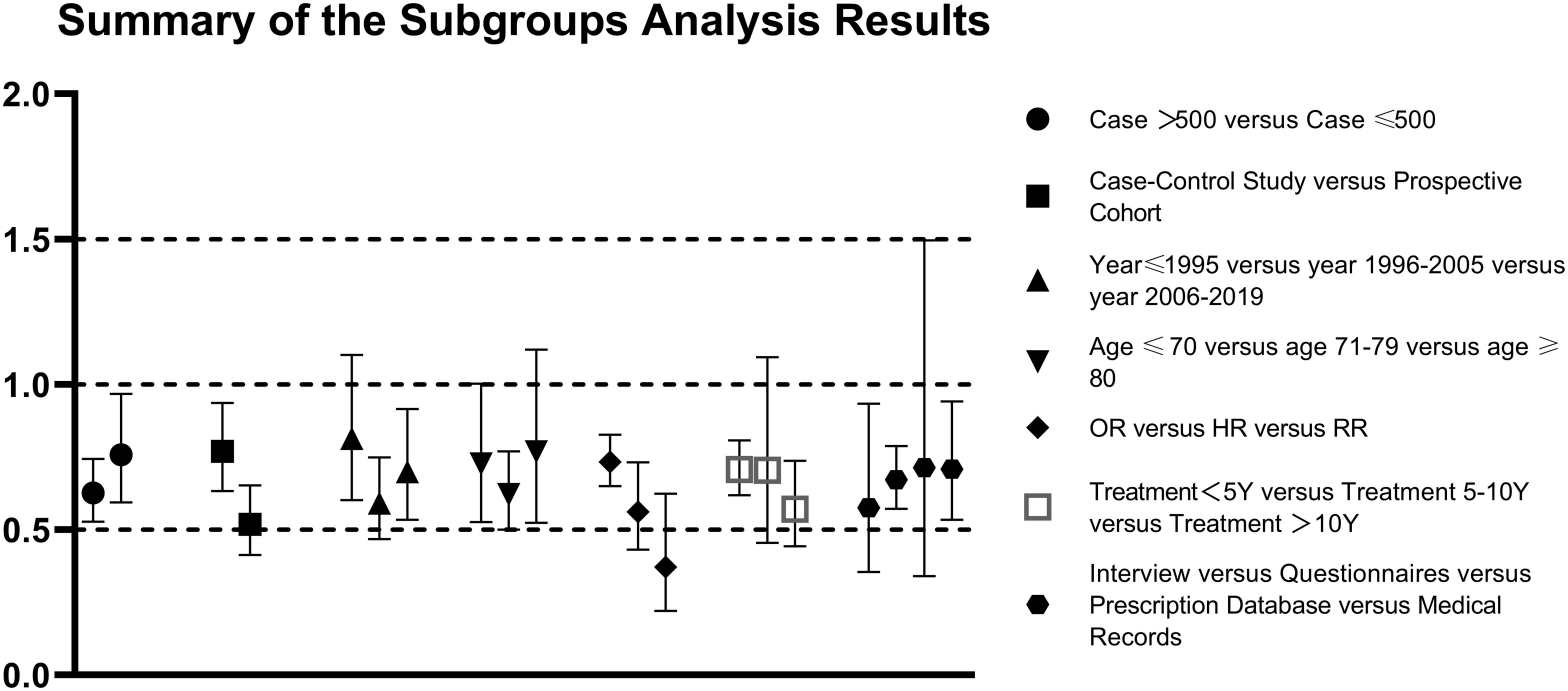
The following subgroups were defined in the Alzheimer's disease (AD) group: case >500 vs. case ≤500,case-control study vs. prospective cohort, publish year ≤ 1995 vs. 1996–2005 vs. 2006–2019, women age ≤70 vs. 71–79 vs. age ≥80, measure of effect = odds ratio (OR) vs. hazard ratio (HR) vs. relative risk (RR), hormone therapy ascertainment by interview vs. questionnaires vs. prescription database vs. medical records, duration of the treatment <5 years vs. 5–10 years vs. treatment >10 years.

**Figure 4:**
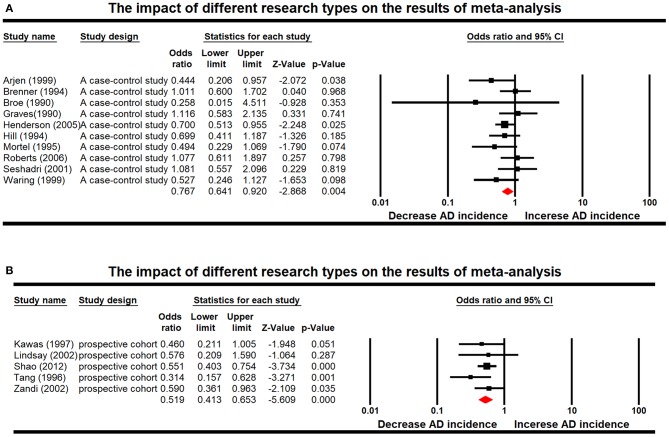
Forest plot displaying random-effects meta-analysis results for the impact of different research types, which were case-control study **(A)** and prospective cohort **(B)**.

Furthermore, according to the results of subgroup analyses in the AD group, heterogeneity came from data measure. Two of the interaction terms of the predefined subgroups showed statistical significance: study design (*P* = 0.01) and measure of effect (*P* = 0.03). Estimated pooled differences among each subgroup are presented in Figure 3. For the stratified analyses among studies of AD, different measure of effects are the source of heterogeneity, but the root cause is different in effect design, suggesting that we should classify different research types before statistical analysis in meta-analysis. Forest plot displayed random-effects meta-analysis results for different effect designs in the AD subgroups (Figure 4). When only prospective cohort studies were included, we observed an increased effective size for AD studies (OR: 0.519; 95% CI: 0.413–0.653; *P* < 0.001), adding more proof that ERT is indeed beneficial for treating AD.

### Sensitivity Analyses

**Figure 5:**
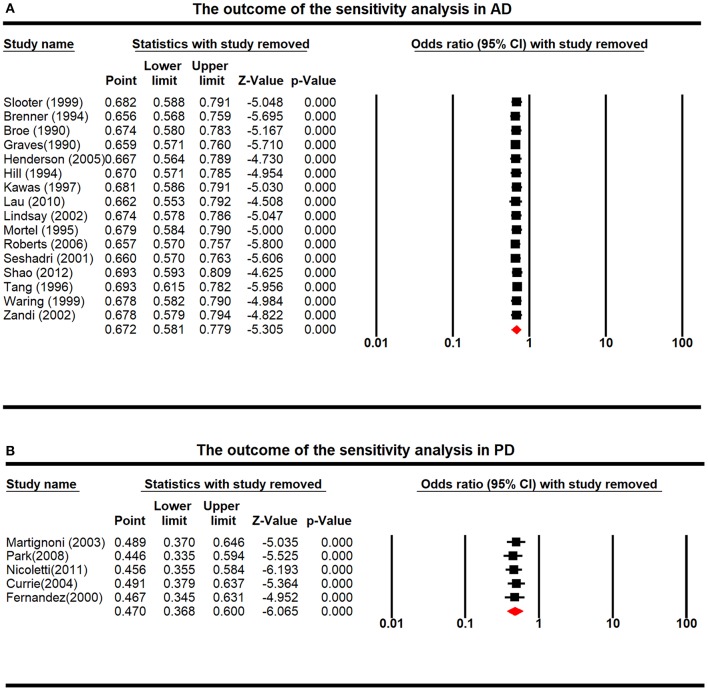
The outcome of the sensitivity analysis in Alzheimer's disease (AD) **(A)** and Parkinson's disease (PD) **(B)**, with the exclusion of one study.

Sensitivity analysis demonstrated that none of the individual studies could induce statistical bias regarding the association between ERT and incidence of AD or PD, indicating that our findings were statistically reliable (Figure 5).

### Publication Biases

**Figure 6:**
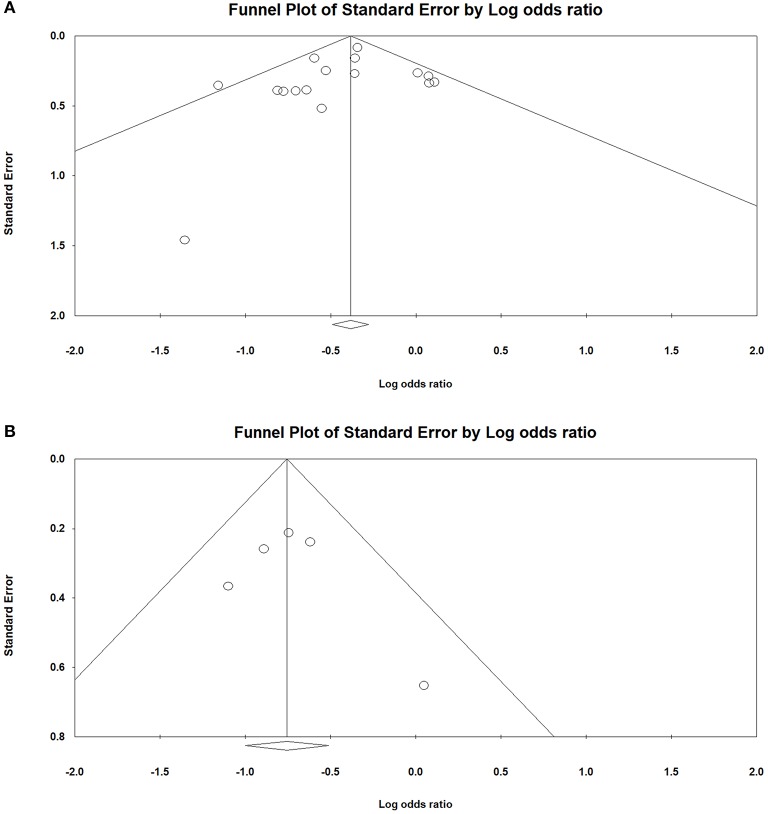
The funnel plot was symmetrical in Alzheimer's disease (AD) **(A)** and Parkinson's disease (PD) **(B)**, suggesting that there was no publication bias in the current analysis.

Funnel plots were used to assess publication biases. We did not find an obvious asymmetry of funnel plots in any of the comparisons, which suggested that our findings were unlikely to be impacted by severe publication biases (Figure 6).

## Discussion

In this review, we presented results from a series of data on postmenopausal hormone therapy in relation to the risk of onset and/or developing AD and PD. Given the results of meta-analysis and subgroup analysis of our collected data, ERT shows a positive effect on the treatment of AD. The situation is similar in the case of ERT and PD. ERT induces some heterogeneity in the study of AD and can be attributed to the study design. Age, year of publication, number of cases, hormone therapy ascertainment, duration of the treatment, and route of administration do not significantly affect the outcome of the meta-analysis.

### Multifactorial Bias and Time Limit

ERT and neurodegenerative disease performance were related to factors such as age, country, socioeconomic status, and health status. It was not feasible to eliminate these factors from the epidemiological study. Such confounding factors can be the source of some analysis bias. First, there was a big challenge in selecting data since most studies did not report effects in a manner that allowed their results to be used for meta-analyses. There are differences in the determination of estrogen treatment results, treatment duration, or disease interval evaluation in the included articles. We added meta-regression analysis, which showed they were minimally relevant to the result in these subgroups. However, it would indeed become a source of limitation. Second, many of the observational studies showed a clear time-dependent pattern. The data included in this article have a large time span, thus the diagnostic criteria may change. To avoid this bias, we adopted NINCDS-ADRDA diagnostic criteria in AD studies and MDS-UPDRS in PD studies. Compared with other diagnostic criteria, they have been applied for more than 20 years since it was established. At the same time, we also used meta-regression to evaluate whether the diagnostic criteria have a trend of change with time, and the regression result is non-existent. The large controlled studies currently underway will hopefully address this time limit. Third, determining the history of hormone therapy use was a concern in studies that relied on self-report (e.g., interview or questionnaire). Pathological cognitive changes had been a big challenge to recall the memory of hormone therapy use. We included several studies with hospital records or multidisciplinary evaluations on top of patient's self-report to reduce the chance of recall bias.

### Discussion of Subgroup and Regression Analysis

Different researchers used appropriate study designs to study the relationship between ERT and AD and PD. Meta-regression results showed that the study design was indeed the heterogeneous source of meta-analysis (*P* = 0.01). Therefore, we classified the articles into case-control study and prospective cohort study according to different study designs and then re-conducted meta-analysis. When only prospective cohort studies were included, we observed an increased effective size for AD studies (OR: 0.519; 95% CI: 0.413–0.653; *P* < 0.001), adding more proof that ERT is indeed beneficial for treating AD (Figure 4). Therefore, we believe that this study design is more reasonable and effective in the epidemiological study of ERT and neurodegenerative diseases. We call on researchers to be more inclined to choose this research design in future epidemiological studies.

Some literature suggests that the role of ERT may depend on the age of menopause and the therapeutic intervention used. The time window of estrogen therapy is associated with the risk of onset and/or developing neurodegenerative disease, and early treatment performed 10 years after menopause can decrease the risk (Yaffe et al., 1998). Regression analysis for age (*P* = 0.98581 > 0.05) showed no statistical significance. According to the subgroup analysis among age ≤70 (*I*^2^ = 33.396, *P* = 0.199), age 71–79 (*I*^2^ = 38.876, *P* = 0.133), and age ≥80 (*I*^2^ = 17.911, *P* = 0.296), there was no evidence indicating that age was associated with the risk of disease development.

What requires further investigation is the relationship between the route of administration of estrogen therapy and the risk of onset and/or developing neurodegenerative disease. Four studies differentiated the use by route of administration (oral vs. transdermal), as shown in Table 5. Meta-analysis results of the oral route were OR: 0.925, 95% CI: 0.618–1.385, and *P* = 0.707. Results of the transdermal drug delivery were OR: 0.975, 95% CI: 0.731–1.299, and *P* = 0.861. There was no statistical significance between the use of oral estrogens and transdermal estrogens. However, our sample size of only four studies might cause a bias in the result. There was a lack of evidence from large and randomized clinical trials that examine the efficacy and safety of alternative hormone therapy for the route of administration.

**Table 5:** Summary of results–route of administration and Alzheimer's disease risk.

**References**	**Study design**	**No**.	**Covariates**	**Route of administration**	**OR/RR/HR**	**95% CI**
Brenner et al., 1994	A case-control study	227	Education, marital status, ethnicity, smoking or progestogen use	Oral Transdermal	0.70 1.30	0.10–1.50 0.70–2.30
Paganini-Hill and Henderson, 1994	A case-control study	355	Age, weight, stroke, blood pressure, medication use	Oral	0.70	0.50–0.98
				Transdermal	0.48	0.24–0.94
Seshadri et al., 2001	A case-control study	280	Age, smoking, BMI, physician's practice	Oral	0.89	0.35–2.30
				Transdermal	0.73	0.15–3.57
Imtiaz et al., 2017	A case-control study	8,195	Age, education	Oral	1.14	1.10–1.18
				Transdermal	1.07	0.86–1.34

*OR, odds ratio; RR, relative risk; HR, hazard ratio; BMI, body mass index*.

### Mechanism of Estrogen Therapy

Through different regulatory mechanisms, estrogen affects the conduction of nerve signals and tissue changes in the brain. At the same time, genes associated with neurodegenerative diseases are also shown to be regulated by estrogen (Nilsson et al., 2011; Xing et al., 2013), and these results are in agreement with the results of our meta-analysis. It has been reported that estrogen decreases reactive oxygen leak and diffusion lipid peroxidation coupled with oxidative stress and endogenous oxidative damage by increasing electron transport chain complex IV and mitochondrial reactivity (Irwin et al., 2008). Brain-derived neurotrophic factor (BDNF) gene contains an estrogen response element (ERE), which confirms that ERβ affects the maturation and plasticity of synapses through the BDNF-TrkB signaling pathway (Zhao et al., 2011). We have shown that there is an important interaction between the apolipoprotein E (Apo E) gene and the risk of onset and/or developing AD (Liu et al., 2013). ERE presents on the Apo E gene, which can modify the expression of the Apo E gene in the cerebral cortex by 17β-estradiol (Struble, 2003). PD is a neurodegenerative disease caused by substantia nigra degeneration or loss of dopaminergic neurons. It has been found that estrogen can convert D2 DA receptors from a high affinity state to a low affinity state in monkeys with different dyskinesias. An important interaction between the brain renin-angiotensin system (RAS) and effects of 17β-estradiol in models of PD, the RAS enhances the progression of dopaminergic degeneration by intensifying neuroinflammation, and estrogen protects dopaminergic neurons by inhibition of RAS (Labandeira-Garcia et al., 2016). In the PD model, 17β-estradiol is a negative regulator of the RAS, which inhibits its function and reduces neuroinflammation and DA degeneration. Estrogen rapidly and directly acts on striatum and nucleus accumbens, via a G-protein-coupled external membrane receptor, to enhance DA releases and DA-mediated behaviors (Becker, 1999). At the same time, 17β-estradiol is found to inhibit 1-methyl-4-phenyl-1,2,3,6-tetrahydropyridine (MPTP)-induced DA depletion under a dosing regimen (repeated daily administration) (Ramirez et al., 2003). At present, DA agonists are one of the main drugs for symptomatic treatment of PD. It is determined that the beneficial effects of estrogen on DA receptors can delay the progression of PD.

In conclusion, a meta-analysis was conducted with regard to the long-standing debate about whether ERT protects cognition and reduces the risk of neurodegenerative disease. First, different diseases were classified. In the case of AD, more research data were included, beneficial conclusions were thus obtained, which also verified the clinical observation data. Meta-analysis in the estrogen therapy and the risk of PD were first conducted, the results showed that estrogen therapy significantly reduced the risk of PD. These data can help with the development of new therapeutic ideas and preventative measures for future clinical application regarding the development AD and PD.

Some of the minor issues that have been experienced so far with estrogen use were addressed. The results of studies and meta-analysis indicated that estrogen therapy does have beneficial effects on neurodegenerative diseases such as AD and PD. Notably, neurodegenerative diseases are associated with internal energy and material metabolism disorders, which are not limited to reproductive hormones. According to the latest epidemiological studies, neurodegenerative diseases were closely related to diabetes and non-alcoholic fatty liver disease (NAFLD) (Szmuilowicz et al., 2009; Martins, 2014; Slopien et al., 2018; Venetsanaki and Polyzos, 2019). They may have a common pathogenic mechanism, which involves the production of Aβ protein, insulin resistance, and mitochondrial dysfunction (Martins, 2015, 2018). Detection of these endocrine markers that associate with metabolic syndrome would help with timely diagnosis of the disease in the early or presymptomatic phase. Future studies need to determine how the induction or inhibition of endocrinal targets could be used for predictable neuroprotection in neurodegenerative disease therapies.

## Data Availability Statement

All datasets generated for this study are included in the article/supplementary material.

## Author Contributions

QL and YC conceived and designed the study. SL and YS collected the data. YS, XC, and XL analyzed and interpreted the data. YS drafted the manuscript with critical revisions from all the authors.

### Conflict of Interest

The authors declare that the research was conducted in the absence of any commercial or financial relationships that could be construed as a potential conflict of interest.
